# Antagonistic activities of some probiotic lactobacilli culture supernatant on *Serratia marcescens* swarming motility and antibiotic resistance

**Published:** 2017-12

**Authors:** Roya Vahedi-Shahandashti, Rouha Kasra-Kermanshahi, Maliheh Shokouhfard, Parinaz Ghadam, Mohammad Mehdi Feizabadi, Shahram Teimourian

**Affiliations:** 1 Department of Microbiology, Faculty of Biological Sciences, Alzahra University, Tehran, Iran; 2 Department of Biotechnology, Faculty of Biological Sciences, Alzahra University, Tehran, Iran; 3 Department of Microbiology, School of Medicine, Tehran University of Medical Sciences, Tehran, Iran; 4 Thoracis Research Center, Imam Khomeini Hospital, Tehran University of Medical Sciences, Tehran, Iran; 5 Department of Medical Genetics, Iran University of Medical Sciences, Tehran, Iran

**Keywords:** Antibiotic resistance, Probiotics, Swarming, *Serratia marcescens*

## Abstract

**Background and Objectives:**

*Serratia marcescens*, a potentially pathogenic bacterium, benefits from its swarming motility and resistance to antibiotic as two important virulence factors. Inappropriate use of antibiotics often results in drug resistance phenomenon in bacterial population. Use of probiotic bacteria has been recommended as partial replacement. In this study, we investigated the effects of some lactobacilli culture supernatant on swarming, motility and antibiotic resistance of *S. marcescens*.

**Materials and Methods:**

Antimicrobial activity of lactobacilli supernatant and susceptibility testing carried out on *S. marcescens* isolates. Pretreatment effect of lactobacilli culture supernatant on antibiotic - resistance pattern in *S. marcescens* was determined by comparison of the MIC of bacteria before and after the treatment.

**Results:**

Our results showed that pretreatment with *L. acidophilus* ATCC 4356 supernatant can affect the resistance of *Serratia* strains against ceftriaxone, but it had no effect on the resistance to other antibiotics. Furthermore, culture supernatant of lactobacilli with concentrations greater than 2%, had an effect on the swarming ability of *S. marcescens* ATCC 13880 and inhibited it.

**Conclusion:**

Probiotic bacteria and their metabolites have the ability to inhibit virulence factors such as antibiotic resistance and swarming motility and can be used as alternatives to antibiotics.

## INTRODUCTION

*Serratia marcescens* is an important opportunistic pathogen of humans (1). It is a major cause of hospital-acquired pneumonia, urinary tract infections, respiratory tract infections, bacteremia, conjunctivitis, endocarditis, meningitis and wound infections (2–4). *S. marcescens* swarming motility is a surface-associated group behavior that is connected with virulence capability and antibiotic resistance (5, 6). Swarming motility has been noted as the cause of virulence for other Gram-negative bacteria such as *Pseudomonas aeruginosa* (7). *S. marcescens* flagella and surfactants, known as serrawettins, contribute to swarming motility (8–10). *S. marcescens* swarms on Luria-Bertani (LB) agar surfaces at 30°C, but not at 37°C (11). Increased resistance among pathogens, causing nosocomial and community acquired infections, has been attributed to the widespread usage of antibiotics (12). The main problem associated with *S. marcescens* infections is increase in its resistance against various antibiotics (13). Therefore, it is essential to find new therapeutic approach for the treatment of such infections. Preparing prevention and treatment protocols with using natural products seems to be necessary (14). Recent reports have documented the role of *Lactobacillus* in the prevention and treatment of some infections. *Lactobacillus* species and strains live as commensals in the human body (15). Their beneficial effect may be associated to their ability to inhibit the growth of pathogens, apparently by the secretion of antibacterial substances including lactic acid, hydrogen peroxide, etc. (16). Nowadays, application of probiotics for prevention and management of gastrointestinal disorders has received much interest (17). In this study, due to the increasing antibiotic resistance, especially among Gram-negative bacteria the inhibitory effects of several lactobacilli culture supernatants on some *S. marcescens* strains virulence factors was investigated after producing susceptible phenotype as a new way for treating antibiotic resistance pathogens.

## MATERIALS AND METHODS

### Bacterial strains and culture conditions.

*Lactobacillus plantarum* ATCC 8014, *L. acidophilus* ATCC 4356, *S. marcescens* ATCC13880 and *S. marcescens* ATCC 19180 were purchased from Iranian Research Organization for Science and Technology (IROST). The *Lactobacillus* species were grown in the Man, Rogosa, Sharpe Broth (MRSB; Darmstadt, Merck, Germany) and incubated at 37°C in an anaerobic jar for 24 h and maintained on MRS agar plates (MRSA; Darmstadt, Merck, Germany). *S. marcescens* strains were grown in Nutrient Broth (NB; Darmstadt, Merck, Germany) and incubated at 37°C for 24 h.

### Antimicrobial activity and nature of antimicrobial substances in lactobacilli supernatant.

The inhibitory activity of supernatants of *L. acidophilus* and *L. plantarum* was screened against *S. marcescens* strains using Micro scale Optical Density Assay (MODA) (18). Cell-free culture supernatants (CFCS) were obtained by centrifugation (13,000 × g, 4°C and 15 min) of *L. acidophilus* and *L. plantarum* cultures grown in 20 ml MRS broth at 37°C for 24 h. The supernatant was filtered through a 0.22 mm filter to remove cells, and then 1 ml CFCS of *L. acidophilus* and *L. plantarum* was retained as untreated filtrate. To determine the effect of organic acids, 1 ml CFCS was adjusted to pH 7. The neutralized CFCS was then treated with catalase (5 mg ml^−1^, Sigma) at 25°C for one h to eliminate the possible inhibitory effect of H_2_O_2_. Pepsin and trypsin sensitivity was evaluated by incubating one ml CFCS with proteolytic enzymes, including Pepsin (1 mg ml^−1^, Sigma) and Trypsin (1 mg ml^−1^, Sigma) at 37°C for 2 h. Briefly, in a 96 well plate, 100 μl of diluted (1:10,000 in NB) test culture was added. Triplicate wells for each test culture, one well which nothing was added (no supernatant or media) just 100 μl of diluted test culture; one well, served as a negative control, in which 15 μl of MRS was added; and the third well served as the test well to which 15 μl of cell-free *L. plantarum* or *L. acidophilus* treated supernatant (with NaOH, H_2_O_2_, Pepsin and Trypsin) were added. Each series was run in duplicate on the same plate. The plate was then incubated at 37°C for 24 h. After incubation, plates were read using a micro plate reader at 600 nm. The difference in absorbance between control (media) and samples were used to report antibacterial activity as percent difference in cell growth (18).

### Susceptibility testing.

The minimum inhibitory concentrations (MICs) were determined according to the clinical laboratory Standards Institute (CLSI) guideline (2015) using micro titer plate method. In this method, colonies of lactobacilli from TSA were suspended in Muller Hinton broth (Merck, Germany) and the turbidity of suspension was adjusted to 0.5 McFarland and subsequently diluted in Muller Hinton broth (1:100) to reach a final concentration of 1 × 10^6^ CFU/ml. Dilutions of cephalothin (Sigma-Aldrich), cefazolin, amikacin, ceftriaxone and ceftazidime (Exir, Broujerd, Iran) were made in distilled water. The antibiotics were prepared at different concentrations ranged from 0.125 to 512 μg/ml. Each well was filled with 100 μl of each dilution of the antibiotic and 100 μl of bacterial suspension. Each plate included positive controls (bacteria without an antimicrobial), negative controls (medium only). Micro titer plates were incubated at 37°C for 24 h. MIC was determined as the minimum antibiotic concentration that inhibited the visible growth (19). All tests were carried out in duplicates.

### Determination of MIC of lactobacilli supernatant against *S. marcescens* strains.

The MIC of the supernatant was determined according to Wikler et al. (2015) method with some modifications (19). In brief, the highest and the lowest concentrations were 250 μg/ml and 0.5 μg/ml respectively. Moreover, the supernatant was directly added to the wells. A stock solution of supernatant was prepared in sterile Muller-Hinton broth (256 μg/ml) which was further diluted in MHB to reach concentration range of 0.5 μg/ml to 256 μg/ml. Afterwards, 100 μl culture of one of the test bacteria, grown to the early stationary growth phase in MHB, was added to 1 ml of MHB in tube and final concentration of bacteria in individual tubes was adjusted to about 5 × 10^6^ CFU/ml. Control tubes contained; only culture media without any antibacterial agent, culture media with pathogenic strains (5 × 10^6^ CFU/ml), and culture media with supernatant. After 24 h incubation at 37°C, the MIC was determined as lowest concentration that could inhibit visible bacterial growth for 24 h (20, 21).

### Pretreatment effect of lactobacilli culture super-natant on antibiotic-resistant pattern in *S. marcescens.*

The MICs of lactobacilli culture supernatant were determined as explained above. Then *S. marcescens* strains were cultured in sub-MIC (1/2 MIC) concentrations of the supernatant. After incubation at 37°C for 18 h, bacteria were cultured in LB broth medium and were incubated until achieving 0.5 Mc-Farland standards and then the amount of MIC was determined for antibiotics according to Wikler et al. (19). Finally, the MIC of antibiotics for bacteria was compared before and after the treatment (22, 23).

### Assay of swarming inhibition.

Standard NCCLS agar dilution method was used to test the anti-swarming activity of lactobacilli supernatant (24). *L. acidophilus* ATCC 4356 and *L. plantarum* ATCC 8014 were cultured in the Man, Rogosa, Sharpe Broth or agar and incubated at 37°C in an anaerobic jar for 24 h. The supernatant of overnight cultures of *L. acidophilus* and *L. plantarum* was separated and neutralized by catalase (5 mg/ml^−1^) and trypsin (1mg/ml^−1^). Various concentrations of supernatants (ranging from 0.5% to 4% v/v) were added to the PG (pep-tone glycerol [peptone, 5 g/liter; glycerol, 1% v/v; agar-agar 0.7%]) (25). Plates were inoculated with 3 μl (10^8^ CFU/ml^−1^) of *S. marcescens* ATCC 13880, and incubated at 30°C. Growth and swarming were monitored after 24 h. The supernatant neutralized by NaOH (1N), catalase (5 mg/ml^−1^) and trypsin (1mg/ml^−1^), then used as the control of growth (26).

### Statistical analysis.

SPSS version 20 was used for statistical analysis. The result of triplicate experiments was averaged, and significance level was set at P < 0.05. Then one way analysis of variance (ANOVA) was performed for comparing between groups.

## RESULTS

### The antimicrobial activity and nature of antimicrobial substances of lactobacilli supernatant.

**Fig. 1.**
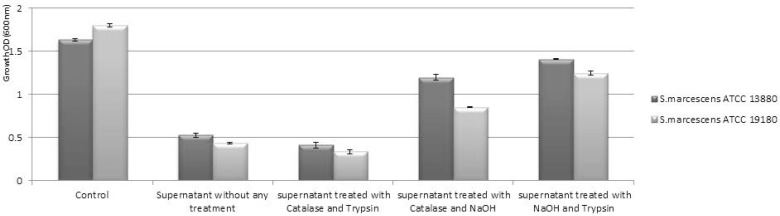
MODA of cell-free supernatant from *L. acidophilus* ATCC 4356 on *S. marcescens* strains growth.

**Fig. 2.**
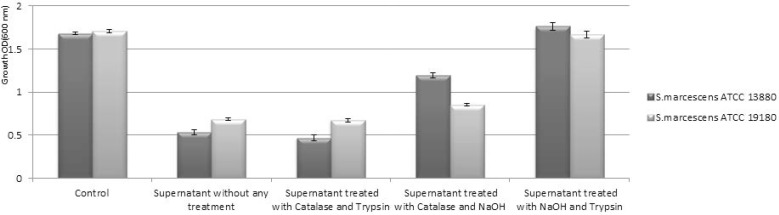
MODA of cell-free supernatant from *L. plantarum* ATCC 8014 on *S. marcescens* strains growth.

The data presented in Figs. 1 and 2 show the inhibitory activity of the *L. plantarum* and *L. acidophilus* supernatants measured by MODA. Comparison of the non-treated supernatant of both strains with that of the control (MRS medium) revealed an inhibitory effect of the supernatant from both strains on *S. marcescens* ATCC 13880 and *S. marcescens* ATCC 19180. Then the supernatant of both strains of *Lactobacillus* were neutralized with catalase, trypsin, and NaOH compared with the control. The results indicated that organic acids and proteinaceous components had a significant role in the antimicrobial activity of the supernatant (p < 0.05), but the neutralized supernatant of both strains of lactobacilli with NaOH and trypsin had no significant antimicrobial activity (p > 0.05).

### Determination of MICs.

The MIC breakpoint of ≥ 4μg/ml considers as resistance of bacterium to cefazolin and ceftriaxone and the MIC breakpoint of ≤ 1μg/ml indicates the susceptibility of bacterium to these antibiotics (Table 1). The MIC breakpoint of ≥ 16μg/ml, ≥ 64μg/ml and ≥ 32μg/ml shows ceftazidim, amikacin and cephalotin resistance respectively in the bacterial strain and the MIC breakpoint ≤ 4μg/ml, ≤ 16μg/ml and ≤ 8μg/ml explained the susceptibility to these antibiotics respectively. Consequently, both *S. marcescens* strains were resistant to cephalotin, ceftazidim, ceftriaxon but susceptible to Amikacin. On the other hand, *S. marcescens* ATCC 13880 was resistant and *S. marcescens* ATCC 19180 was susceptible to cefazolin.

**Table 1.** Minimum Inhibitory Concentrations (MICs) of antibiotics against *S. marcescens* strains (0.125 to 512 μg/ml)

**Antibiotic**	**MIC breakpoint (μg/ml)**	

	***S. marcescens*** **ATCC 13880**	***S. marcescens*** **ATCC 19180**
Cephalothin	512	512
Cefazolin	64	0.125
Amikacin	1	0.125
Ceftriaxone	128	64
Ceftazidime	512	512

### Determination of MIC of lactobacilli supernatant against *S. marcescens* strains.

MIC of cell free supernatant of *Lactobacillus* strains has been shown in Table 2.

**Table 2.** Minimum Inhibitory Concentrations (MICs) of lactobacilli supernatant against *S. marcescens* strains (0.5 μg/ml to 256 μg/ml)

**Cell free supernatant of *Lactobacillus* strains**	**MIC breakpoint (μg/ml)**	

	***S. marcescens* ATCC 13880**	***S. marcescens* ATCC 19180**
*L. plantarum* ATCC 8014	64	32
*L. acidophilus* ATCC 4356	16	8

### The effect of lactobacilli culture supernatant pretreatment on antibiotic-resistant pattern in *S. marcescens*.

Table 3 shows the MICs of treated *S. marcescens* strains with sub-MIC concentrations of each lactobacilli culture supernatants. It was found that *L. acidophilus* and *L. plantarum* culture super-natant treated strains of *S. marcescens* remained unchanged to cephalothin, cefazolin, amikacin and ceftazidime compared with non-treated. While, the sensitivity of *S. marcescens* strains to ceftriaxone after treatment with *L. acidophilus* supernatant changed.

**Table 3.** Minimum Inhibitory Concentrations (MICs) of antibiotics against *S. marcescens* strains after 24 h treatment with lactobacilli culture supernatant (0.125 to 512 μg/ml)

**Antibiotic**	***S. marcescens* ATCC 13880**		***S. marcescens* ATCC 19180**	

	**Treated with *L. acidophilus* Supernatant**	**Treated with *L. plantarum* Supernatant**	**Treated with *L. acidophilus* Supernatant**	**Treated with *L. plantarum* Supernatant**
Cephalothin	512	512	512	512
Cefazolin	64	64	0.125	0.125
Amikacin	1	1	0.125	0.125
Ceftriaxone	64	128	32	64
Ceftazidime	512	512	512	512

### Effects on swarming motility.

**Fig. 3.**
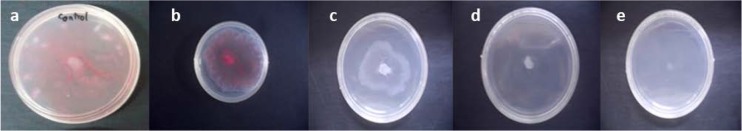
Swarming motilities were assayed in PG (peptone glycerol [peptone, 5 g/l; glycerol, 1% v/v; agar-agar 0.7%]) containing concentrations of *L. acidophilus* ATCC 4356 supernatant ranging from 0.5% to 4% v/v. Plates were photographed after 24 h of incubation at 30°C. a) Control contained treated supernatant with NaOH and Trypsin. b, c, d and e) test containing 0.5%, 1%, 2% and 4% of supernatant, respectively.

**Fig. 4.**
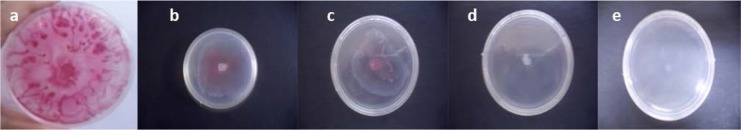
Swarming motilities were assayed in PG (peptone glycerol [peptone, 5 g/liter; glycerol, 1% v/v; agar-agar 0.7%]) containing concentrations of *L. plantarum* ATCC 8014 supernatant ranging from 0.5% to 4% v/v. Plates were photographed after 24 h of incubation at 30°C. a) Control contained treated supernatant with NaOH and Trypsin. b, c, d and e) Test containing 0.5%, 1%, 2% and 4% of supernatant, respectively.

Treated supernatant of *L. acidophilus* ATCC 4356 and *L. plantarum* ATCC 8014 with NaOH and Trypsin had no effect on swarming motilities (Figs. 3 and 4). While concentrations of 4% and 2% of supernatant of both strains of lactobacilli without treating, completely inhibited swarming motility. Swarming inhibition by 1% v/v was more apparent than by 0.5% v/v and the control plate showed no inhibition of swarming.

## DISCUSSION

In recent decades, worldwide overuse and non-prudent use of antibiotics are leading to the global health care issue of antibiotic resistance. Resistance to common antibiotics in the treatment of nosocomial infection caused by bacteria has increased and created serious problems in the treatment of these diseases. *S. marcescens* is a growing problem for public health, because of its high resistance against many antibiotics and its increasing role in hospital acquired infections. It is important to prevent the spreading of bacteria from patient to patient (27). *S. marcescens* infections have high resistance against some cephalosporins, aztreonam and imipenem (28). In this research, the MICs of cephalosporins and amikacin against *S. marcescens* strains were measured (Table 1). Then, the MICs of antibiotics were measured after treating the *Serratia* strains with sub-MIC concentrations of lactobacilli supernatants (Table 3). According to our results, it can be deduced that *L. acidophilus* supernatant was able to change the antibiotic resistance patterns of *S. marcescens* strains against ceftriaxone but had no effect on the other antibiotics resistance pattern. Treatment with *L. acidophilus* supernatant reduced the resistance of both strains of *Serratia* to ceftriaxone. Ceftriaxone inhibits the mucopeptide synthesis of the bacterial cell wall. In one study by Alakomi et al. (2000), it was found that lactic acid produced by *Lactobacillus* strains can increase the susceptibility of Gram-negative bacteria to the antimicrobial agents (28). Such effect on ceftriaxone resistance may be associated with lactic acid or proteinaceous component of the lactobacilli supernatants on bacterial cell wall permeability and there was an indirect relationship between the pH value of lactobacilli supernatants and penetration of antibiotics into the bacterium. Similar work was done in this field by Naderi et al. (2014), who found that pretreatment with lactobacilli supernatants could be effective on some antibiotic resistant Gram negative bacteria such as *E. coli*, but not *Klebsiella* spp and *Entrobacter* (22). In another study by Shahriar et al. (2012), pretreatment with Sodium Dodecyl Sulfate (SDS) and acridine orange did not affect the antibiotic resistance patterns and plasmid isolation of *Klebsiella* spp (23). Taylor et al. (2002) suggested that the use of agents that do not kill pathogenic bacteria, but modify them to produce a susceptible phenotype to antibiotic could be an alternative approach to the treatment of infectious disease (29). Such agents could render the pathogen susceptible to a previously ineffective antibiotic, and because the modifying agent applies little or no direct selective pressure, this concept could slow down or prevent the emergence of resistant genotypes. The search for solutions to the global problem of antibiotic resistance in pathogenic bacteria has often focused on the isolation and characterization of new antimicrobial compounds from a variety of sources such as probiotic bacteria. Large varieties of compounds produced by lactobacilli have proved to have therapeutic potentials as antimicrobials and as resistance modifiers.

*S. marcescens* is an important nosocomial pathogen that possesses a repertoire of virulence factors and displays multicellular behavior such as swarming and biofilm formation. Swarming of *Serratia* has been implicated in pathogenesis (11, 28). The increasing evidence of antibiotic resistance requires developing alternative strategies for treatment. This study aimed to find lactobacilli supernatant with antibacterial potentials in order to control antibiotic resistant pathogens. We showed that treated supernatant of both lactobacilli strains with trypsin and catalase has the ability to inhibit *S. marcescens* swarming significantly in concentration greater than 2% and inhibited swarming completely at 4%. But concentration less than 1% had no effect on swarming motility (Figs. 3 and 4). On the other hand, treated supernatant with Trypsin and NaOH significantly affect the growth of both *Serratia* strains (Figs. 1 and 2). The results indicate that organic acid and proteinaceous components both had effect on growth of *Serratia* while NaOH neutralized super-natant had no effect on swarming. This confirms direct impact of the organic acids of the supernatant. Similar works with similar results have also been reported by Roshid et al. (2014), Inoue et al. (2008) and Ghaidaa et al. (2013), who found several agents such as: plants extract, fatty acids and p-nitrophenylglycerol are effective on swarming of pathogenic bacteria including *Proteus* and *P. aeruginosa* (30–32).

It is now well known that many bacterial functions including swarming, biofilm formation, and secretion of virulence factors that are important in successful and recurrent establishment of bacterial infections are related to quorum sensing (QS) (33, 34). Thus, inhibiting QS or anti-QS is an important anti-infectious measure that does not rely on antibiotics (35). Anti-QS agents will inhibit QS mechanism, attenuate virulence determinants and are unlikely to cause drug-resistance related problems (36). With the appearance of multi antibiotic-resistant bacteria; it is becoming increasingly more difficult to treat bacterial infections with conventional antibiotics. Thus, there is an increasing need for new strategies to cope with infectious diseases. It has been suggested that inactivating the QS system of a pathogen can result in a significant decrease in virulence factor production (37–39). So, the possible mechanism by which supernatant of lactobacilli could inhibit *S. marcescens* swarming and virulence factor expression may be due to its acting as an inhibitor compound for bacterial quorum sensing (40, 41).
